# Heat stress tolerance indices for identification of the heat tolerant wheat genotypes

**DOI:** 10.1038/s41598-023-37634-8

**Published:** 2023-07-05

**Authors:** Kavita Lamba, Mukesh Kumar, Vikram Singh, Lakshmi Chaudhary, Rajat Sharma, Shikha Yashveer, M. S. Dalal

**Affiliations:** 1 Department of Genetics and Plant Breeding, Chaudhary Charan Singh Haryana Agricultural University, Hisar, Haryana 125 004 India; 2 Department of Molecular Biology, Biotechnology and Bioinformatics, Chaudhary Charan Singh Haryana Agricultural University, Hisar, Haryana 125 004 India

**Keywords:** Plant breeding, Plant stress responses

## Abstract

Heat stress is one of the major challenges in wheat cultivation because it coincides with the flowering period and limits the crop productivity. This study was conducted for evaluation of 50 wheat genotypes to identify the heat stress tolerant genotypes for improvement of stress tolerance. All genotypes were cultivated for two consecutive years (2018–2020) under normal and late sown conditions. The results of the study revealed that the combined analysis of variance indicated significant variations among genotypes for all the studied stress indices. The reduction in mean grain yield of all genotypes under stress condition as compared to non-stress condition, indicating that the heat stress significantly affect the grain yield. The correlation analysis showed that the negative correlation of tolerance index and stress susceptibility percentage index with the grain yield of genotypes under heat stress condition (Ys) and a highly positive correlation of stress tolerance index, mean productivity, geometric mean, harmonic mean and mean relative performance with grain yield (Yp and Ys) under both conditions, helped accurately to identify the desirable genotypes. From the results obtained from principal component, biplot and cluster analysis, it was reported that HD 2967, WH 1249, HI 1617, WH 1202, WH 1021 and WH 1142 are suitable and good yielding genotypes under both conditions. Thus, above genotypes can be used for cultivation at high temperature or as genetic resources for introducing genetic variations in wheat genotypes to improve stress tolerance.

## Introduction

Wheat (*Triticum aestivum* L.) is one of the world’s foremost food crops, covering more land for farming than any other food crop on the planet. About 1/3rd of the world’s population depends on it for feed and food. Climate change and the growing global population both pose threats to food security^1^. As a result of climate change, there are more extreme temperatures because of less rainfall, changing rainfall pattern, and distribution, as well as shorter winter seasons. The most detrimental abiotic stresses for the growth and development of crop plants, impacting yield potential and the final quality of food items, are drought and heat stress^2^. High temperature stress that limits productivity of important crops including wheat, is likely to increase due to continued change in global climatic conditions^3^. Climate predictions estimate that, by the end of the twenty-first century, the average increase in temperature will be 1–4 °C and will decline the wheat yield by 4.1–6.4%^4,5^. High temperature is also a major constraint on plant growth and development, physiological functions, grain formation and ultimately in yield^6,7^. Additionally, heat stress affects a number of metabolic processes, including protein synthesis, the inactivation of enzymes, and cellular physiological processes, including cell membrane degradation. Heat stress also negatively impacts cell division^2^. In wheat during the anthesis to grain maturity stage, 22–25 °C is optimum temperature beyond this it causes irreversible damage^8^. During late planting of wheat genotypes, their anthesis and grain filling stage influenced by a high temperature of 25–32 °C and this much high temperature causes early maturation of crop resulted in major reduction of grain yield. The world's population is expected to reach 9.1 billion people by the year 2050^9^. Therefore, by developing wheat varieties that are tolerant to high temperatures, its production and productivity can be increased to fulfill the food requirements of the highly growing population^10^. Extreme temperature stress conditions during the crop cycle are the way that climate change has a negative effect on wheat performance. Therefore, it is crucial for breeders to identify genotypes that are resistant to heat. Kamrani et al.^11^ and Khan et al.^12^ also suggested that stress tolerant genotypes could be selected by growing advanced wheat lines under normal and stressed conditions. Exploitation of genetic variation in different wheat genotypes helps the plant breeder to make genotypes stress tolerant. Thus, better selection approach is most challenging for breeders in identification of heat tolerant cultivars.

For the identification of stress tolerant cultivars, several researchers have suggested many stress tolerance indices however, few are more useful for selection of heat tolerant genotypes in wheat. The tolerance index (TOL) was defined as the difference between grain yield under normal and stress conditions^13^. The mean productivity index (MP) is defined as the average yield of genotype under normal (Yp) and stress conditions (Ys)^13^. Fernandez^14^ developed the stress tolerance index (STI), which identifies tolerant genotypes under normal and heat stressed conditions. It is based on the geometric mean production index. The stress susceptibility percentage index (SSPI) was proposed for testing trait stability and differences in traits under both conditions^15^. Fisher and Wood^16^ introduced the relative stress index (RSI) during drought stress in wheat varieties. Farshadfar et al.^17^ used harmonic mean (HM) in on wheat–rye disomic addition lines, as it is the ratio of a doubled product of genotypes yield and their sum under both conditions. The mean relative performance (MRP) is another stress index. The yield stability index (YSI) was calculated as the ratio between the yield under stress and normal conditions^18^. Gavuzzi et al.^19^ introduced the yield index (YI) as the ratio of yield of genotypes under normal and the mean yield of all genotypes under stress conditions. Basavaraj et al.^20^ used percent yield reduction (PYR) in their research on low phosphorus stress in rice. High MP, STI, GMP, HM, YSI and YI values, as well as low values for the TOL, SSI, RSI and PYR are better ways for the stable and tolerant genotypes selection^21^.

In the current study, we have evaluated the 50 wheat genotypes under different environmental conditions (normal and late sown) and observed that at late sown conditions the yield was reduced due to exposure of high temperaure (~ 3–4 °C higher than normal sown) at anthesis and grain filling stages of plants. The eleven stress indices like stress tolerance index (STI), mean relative performance (MRP), harmonic mean (HM), geometric mean (GMP), mean productivity (MP), yield index (YI), yield stability index (YSI), percent yield reduction (PYR), relative stress index (RSI), tolerance index (TOL) and stress susceptibility percentage index (SSPI) have been used for identification and selection of heat tolerant genotypes. Various heat tolerant genotypes were selected on the basis of evaluation of values of stress indices, correlation between grain yield and stress indices by correlation coefficient principal component, biplot and cluter analysis.

## Materials and methods

### Experimental material

The experimental materials were consisted of 50 genotypes (released varieties) of bread wheat (*Triticum aestivum* L). This material is collected from Wheat and Barley Section, Department of Genetics & Plant Breeding, CCS Haryana Agricultural University, Hisar. All genotypes with their pedigree details are presented in Table 1.

**Table 1:** List of all genotypes used in the study with their parentage details.

Sr. No.	Genotypes	Parentage
1	WH 1025	C 591/PBW 231
2	WH 1255	UP 2338/WH 542//PI3043
3	DPW 621–50	KAUZ//ALTAR84/AOS/3/MILAN/KAUZ/4/HUITES
4	WH 1262	92.001E7.32.5/SLVS/5/NS-732/HER/3/PRL/SARA//TSI/VEE#5/4/
		FRET2/6/SOKOLL/3/PASTOR//HXL7573/2*BAU
5	WH 1249	BECARD/FRIVCLN
6	WH 542	JUPATECE/BLUEJA/URES
7	WH 1259	SNB//CMH79A.955/3*CNO79/3/ATTILA/4/CHEN/AEGILOPS
		SQUARROSA (TAUS)//BCN/3/2*KAUZ/5/KINGBIRD#1
8	WH 730	CPAN2092/Improved LOK-1
9	WH 1105	MILAN/S87230//BABAX
10	WH 1142	CHEN AEGILOPS SQUARROSA (TAUS)//FCT/3/2*WEAVER
11	HD 3086	DBW14/HD2733//HUW468
12	WH 283	HD1981/RAJ821
13	WH 1254	P12905/P12907//P12884
14	WH 1218	KA/NAC//TRCH13/VORB
15	PBW 343	ND/VG 1944//KAL//BB/3/YACO'S'/4/VEE#5'S'
16	WH 1257	FRNCLN/3/ND643//2*PRL/2*PASTOR/4/FRANCOLIN#1
17	RAJ 3765	HD2402/VL639
18	HD 2967	ALD/CUC//URES/HD2160M/HD2278
19	HD 3059	KAUZ//ALTAR84/AOS/3/MILAN/KAUZ/4/HUITES
20	DBW 14	RAJ3765/PBW343
21	HI 1617	BAJ#1*2/HUIRIVIS#1
22	WH 1267	WHEAR//2*PRL/2*PASTOR/3/KIRITATI/2*TRCH/4/WHEAR//
		2*PRL/2*PASTOR
23	WH 1261	MUNAL#1/FRANCOLIN#1
24	WH 1124	MUNIA/CHTO/AMSEL
25	WH 1258	CROC_1/AE.SQUARROSA(210)//WBLLI*2/BRAMBLING/3/
		VILLA JUAREZF2009/5/BAV92//IRENA/
26	PBW 752	PBW621/4/PBW343//YR10/6*AVOCET/3/3*PBW343/5/PBW621
27	DBW 71	PRINIA/UP2425
28	WH 1123	NI5663/RAJ3765//K9330
29	WH 1266	MILAN/KAUZ//PRINIA/3/BAV92/4/BAVIS
30	WH 1239	TAM200/PASTOR/TOBA97
31	WH 1184	HD2850/WH147
32	WH 1097	ATTILA/BABAX//PASTOR
33	WH 1260	P12968/WH542
34	WH 1156	TILHI/PASTOR
35	WH 1263	P13043/P13038//P13036
36	WH 1131	MUNIA/CHTO//AMSEL
37	WH 1265	P11906/P11925//P11906
38	HD 3219	PBW343/HD2879
39	WH 1155	SERI*3//RL6010/G*TR/3/PASTOR/4/BHU92
40	C 306	REGENT19473*CHZ//*2C591/3/P19/C281
41	WH 1202	D67.2/PARANA66.270//AE.SQ(320)/3/CUNNINGHAM
42	NBPGR 9	EC178071-270
43	NBPGR 10	EC178071-212
44	DBW 88	KAUZ//ALTAR84/AOS/3/MILAN/KAUZ/4/HUITES
45	WH 1247	SOKOLL/3/PASTOR//HXL7573/2*BHU/4/SOKOLL/WBLLI
46	WH 1240	WBLLI*2/VIVITSI
47	DBW 90	HUW468/WH730
48	WH 711	ALD 'S' HUAC // HD 2285 /3/HFW.17
49	NBPGR 35	IC252653
50	WH 1021	NYOT95/SONAK

### Location, experimental site and environments

The field experiment was carried out in the Research Area of Wheat and Barley Section, Department of Genetics & Plant Breeding, CCS Haryana Agricultural University, Hisar which is situated at latitude of 29°10′N, longitude of 75°46′E and altitude of 215.2 m (705 ft) above sea level in subtropical region of North Western Plain Zone of India. The region belongs to the alluvial plain of GhaggarYamuna and its southern and western portions mark a gradual transition to the desert.

The genotypes were sown in November (November 14, 2018 and November 9, 2019) for normal sown conditions and in December (December 15, 2018 and December 22, 2019) for late sown conditions during *Rabi* season of years 2018–19 and 2019–20 (Table 2).

**Table 2:** Details of the experiments conducted during 2018–2019 and 2019–2020 crop seasons.

Location	Years	Environments	Date of sowing	Date of harvesting	Plant response
Research Area of Wheat and Barley Section, CCS HAU Hisar	2018–19	Normal sown	November 14, 2018	April 21, 2019	Normal conditions
		Late sown	December 15, 2018	April 27, 2019	Heat stress conditions
	2019–20	Normal sown	November 9, 2019	April 25, 2020	Normal conditions
		Late sown	December 22, 2019	May 1, 2020	Heat stress conditions

### Experimental layout

The experiment was laid out in two replications in Randomized Block Design (RBD). When adequate moisture was available, genotypes were seeded in the field. In each replication, each genotypes were grown in 1.2 m^2^ plot with 2 rows, each of 3 m in length. Depending on the rainfall, the field was irrigated at regular intervals and recommended standard cultural and agronomic practices were followed to raise a healthy crop.

### Environmental evaluation

**Figure 1:**
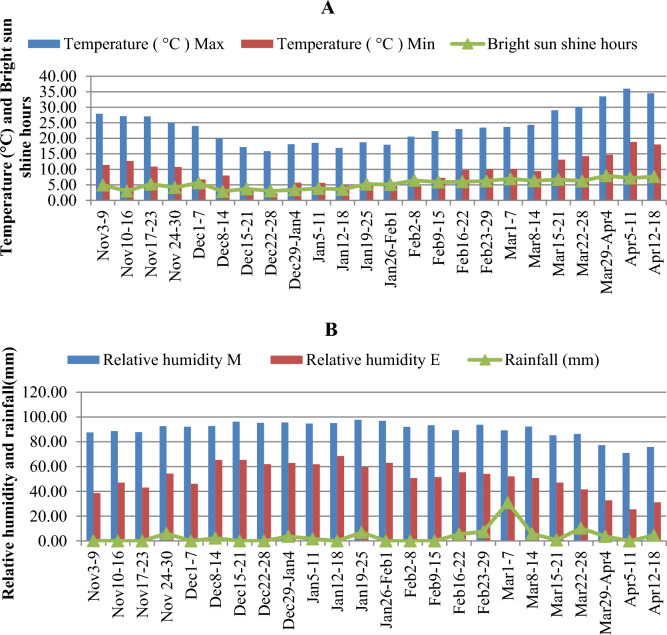
Pooled weekly weather parameters [minimum and maximum temperature (°C), bright sun shine hours (**A**), relative humidity (morning and evening) and rainfall (mm) (**B**)] during wheat growing season (2018–19&2019–20).

The weather parameters were collected from the Department of Agri. Meteorological, CCS HAU, Hisar for two years during crop growing season (November to April) (Fig. 1). The maximum temperature observed was 26.0 °C for normal sown genotypes during anthesis stage while genotypes of late sown conditions faced a temperature of 29.2 °C during 2018–2019. There was a ~ 3 °C rise in temperature at stressed conditions, this high temperature at anthesis filling stage causes early maturation and reduction in grain yield. During this year at anthesis stage the maximum realative humidity was 95%, bright sun shine hours were 6.7 and rainfall was 0.2 mm while during year 2019–20 the anhesis stage of normal sown faced maximum temperature of 20.9 °C, realative humidity of 93%, bright sun shine hours 5.8 and rainfall was 14.8 mm but the plants of stressed conditions had a maximum temperature of 32.6 °C, realative humidity of 81%, bright sun shine hours 7.3 and no rainfall at anthesis stage. The relative humidity (RH) at anthesis stage during 2018–2019 was high due to rainfall while it was low during 2019–2020 at same stage. So late sown genotypes have experienced heat stress during anthesis to grain filling stage. When encountered anthesis and post-anthesis stage, high temperatures [> 28–30 °C]^22^ can dramatically shrink mature grain weight in wheat, lowering yields^23,24^.

At crop maturity, harvested the genotypes from each plot separately and measured their grain yield (g). This grain yield (Yp and Ys) of genotype and mean yield (Xp and Xs) of all genotypes under normal and late sown respectively, was used to calculate all stress indices. Weekly minimum and maximum temperature (°C), relative humidity (morning and evening), bright sun shine hours and rainfall (mm) during wheat growing season at research farm are demonstrated in Fig. 1 and Table S1(Supplementry). The temperature higher than 25 °C during March adversely affects the anthesis and post anthesis stages of late sown genotypes.

The following calculations were used to compute heat tolerance indices:

- Stress tolerance (TOL) = Yp − Ys Rosielle and Hamblin^13^
- Stress tolerance index (STI) = (Yp × Ys)/Xp2 Fernandez^14^
- Stress susceptibility percentage index (SSPI) = Yp − Ys/ 2(Xp) × 100 Moosavi et al.^15^
- Yield index (YI) = Ys/Xs Gavuzzi et al.^19^
- Yield stability index (YSI) = Ys/Yp Bouslama and Schapaugh^18^
- Relative stress index (RSI) = (Yp/Ys)/(Xs/Xp) Fischer and Wood^16^
- Mean productivity (MP) = (Yp + Ys)/2 Rosielle and Hamblin^13^
- Geometric mean productivity (GMP) = √(Ys × Yp) Fernandez^14^
- Harmonic mean (HM) = 2(Yp × Ys)/(Yp + Ys) Bidinger et al.^25^
- Mean relative performance (MRP) = (Ys/Xs) + (Yp/Xp) Ramirez and Kelly^26^
- Percent yield Reduction (PYR) = (Yp − Ys)/Yp × 100 Farshadfar and Javadinia^27^

where Yp and Ys are the yield performance of varieties while, Xp and Xs are the mean yield of all varieties under normal and heat stress conditions, respectively.

Microsoft Excel was used for calculation of stress indices. Variability package of R software with edition number R4.1.2 was used for analysis of variance and correlation coefficient whereas, IBM SPSS Statistics version 26 was used to exploit Principal component analysis (PCA) and the biplot diagrams for identification of tolerant and susceptible genotypes. Hierarchical cluster analysis (between-group linkage) to observe heat tolerant and susceptible genotypes was done using IBM SPSS Statistics version 26.

### Plant material

The plant material used has been comply with relevant institutional, national, and international guidelines and legislation.

## Results

### Pooled ANOVA for grain yield and stress indices

The averaged data of two years were used as there were no significant differences in grain yield and stress tolerance parameters between two studied seasons. The combined ANOVA revealed that the grain yield under normal, heat stress and stress indicators showed significantly substantial (*P* 0.001) variance among wheat genotypes. (Table 3).

**Table 3:** Combined analysis of variance for grain yield under normal (Yp) and stress (Ys) conditions and heat tolerance indices of wheat genotypes.

Source of variation	DF	Mean square												
		TOL	STI	SSPI	YI	YSI	RSI	MP	GMP	HM	MRP	PYR	Ys	Yp
Replication	1	1576.1	0.003	5.19	0.006	0.0016	0.028	726.3	1085.3	1484.3	0.007	15.93	2190.2	50.4
Genotype	49	13,185.6***	0.03***	43.45***	0.02***	0.01***	0.09***	8483.6***	8007.9***	7722.3***	0.06***	114.48***	6854.2***	16,705.8***
Residuals	49	4300.9	0.005	14.17	0.004	0.003	0.03	1514.6	1371.6	1308.5	0.011	33.99	1512.5	3667.2

*STI* stress tolerance index, *MRP* mean relative performance, *HM* harmonic mean, *GMP* geometric mean, *MP* mean productivity, *Ys* grain yield of genotypes under heat stress condition, *YI* yield index, *Yp* grain yield of genotypes under normal condition, *YSI* yield stability index, *PYR* percent yield reduction, *RSI* relative stress index, *TOL* tolerance index, *SSPI* stress susce.

### Stress tolerance indices

In this study, different stress indices, like STI, TOL, SSPI, YSI, YI, RSI, MP, HM, MRP, PYR and GMP, were calculated based on yield under normal conditions and heat stress conditions (Table 4). The highest value for TOL, SSPI, YSI, RSI and PYR belonged to HD 3059 and identified as heat susceptible genotype because it had high grain yield under normal (non-stress conditions) and low grain yield under late sown (heat stress) conditions and this genotype is suitable for normal sown conditions. The genotype NBPGR 9 with low TOL, SSPI, YSI, RSI, and PYR value was considered as more tolerant to heat. This genotypes was found less performing under both conditions. The declined values of these indices as a result of the minimal yield differential between the two conditions.Therefore, low values does not mean high performing and genotype grain yield should be taken in consideration. The genotype HD 2967 had highest value for STI, MP, GMP, HM and MRP. So this was considered as the most stable and productive genotypes among all genotypes under both conditions but lowest value for same stress indices was shown by WH 1097. The highest values for YI belonged to genotypes HI 1617 and WH 1123 but lowest value belonged to WH 1097.The genotypes with highest value of STI, MP, GMP, HM and MRP were identified as heat tolerant while WH 1097 with lowest value of these indices was treated as heat susceptible genotype. The percent yield reduction was found least in genotype NBPGR 9 followed by WH 1266, HI 1617, WH 1202 and WH 1025, these genotypes showed less yield difference under both conditions.

**Table 4:** Grain yield/plot (g), Yp and Ys under normal and stress conditions and stress tolerance indices of different wheat genotypes.

Sr. No	Genotypes	Yp	Ys	TOL	STI	SSPI	YI	YSI	RSI	MP	GMP	HM	MRP	PYR
1	WH 1025	828.63	631.00	197.63	0.669	11.18	1.06	0.76	1.94	729.81	723.09	716.43	1.99	23.85
2	WH 1255	946.50	650.25	296.25	0.787	16.75	1.09	0.69	2.15	798.38	784.51	770.89	2.16	31.30
3	DPW 621–50	750.63	529.50	221.13	0.508	12.50	0.89	0.71	2.10	640.06	630.44	620.96	1.74	29.46
4	WH 1262	914.50	606.88	307.63	0.710	17.40	1.02	0.66	2.23	760.69	744.97	729.59	2.05	33.64
5	WH 1249	1020.63	664.88	355.75	0.868	20.12	1.11	0.65	2.27	842.75	823.76	805.21	2.27	34.86
6	WH 542	827.88	492.50	335.38	0.522	18.97	0.82	0.59	2.49	660.19	638.54	617.59	1.76	40.51
7	WH 1259	850.63	595.00	255.63	0.647	14.46	1.00	0.70	2.12	722.81	711.42	700.21	1.96	30.05
8	WH 730	922.38	565.38	357.00	0.667	20.19	0.95	0.61	2.41	743.88	722.14	701.04	1.99	38.70
9	WH 1105	866.63	527.50	339.13	0.585	19.18	0.88	0.61	2.43	697.06	676.12	655.82	1.86	39.13
10	WH 1142	984.63	670.63	314.00	0.845	17.76	1.12	0.68	2.17	827.63	812.60	797.84	2.24	31.89
11	HD 3086	939.38	682.50	256.88	0.820	14.53	1.14	0.73	2.04	810.94	800.70	790.60	2.20	27.35
12	WH 283	843.75	515.38	328.38	0.556	18.57	0.86	0.61	2.42	679.56	659.43	639.89	1.82	38.92
13	WH 1254	846.50	635.00	211.50	0.688	11.96	1.06	0.75	1.97	740.75	733.16	725.65	2.02	24.99
14	WH 1218	853.38	627.13	226.25	0.685	12.79	1.05	0.73	2.01	740.25	731.56	722.96	2.01	26.51
15	PBW 343	831.38	548.38	283.00	0.583	16.00	0.92	0.66	2.24	689.88	675.21	660.85	1.86	34.04
16	WH 1257	999.25	637.13	362.13	0.814	20.48	1.07	0.64	2.32	818.19	797.90	778.12	2.20	36.24
17	RAJ 3765	870.75	652.50	218.25	0.727	12.34	1.09	0.75	1.97	761.63	753.77	745.99	2.08	25.06
18	HD 2967	1108.38	678.88	429.50	0.963	24.29	1.14	0.61	2.42	893.63	867.44	842.02	2.39	38.75
19	HD 3059	1053.63	564.38	489.25	0.761	27.67	0.94	0.54	2.76	809.00	771.13	735.03	2.14	46.43
20	DBW 14	757.50	556.25	201.25	0.539	11.38	0.93	0.73	2.02	656.88	649.12	641.46	1.79	26.57
21	HI 1617	893.63	700.63	193.00	0.801	10.91	1.17	0.78	1.89	797.13	791.26	785.44	2.18	21.60
22	WH 1267	902.25	637.88	264.38	0.736	14.95	1.07	0.71	2.09	770.06	758.63	747.37	2.09	29.30
23	WH 1261	945.13	611.63	333.50	0.739	18.86	1.02	0.65	2.29	778.38	760.30	742.65	2.09	35.29
24	WH 1124	954.00	629.13	324.88	0.768	18.37	1.05	0.66	2.24	791.56	774.72	758.23	2.13	34.05
25	WH 1258	872.00	578.13	293.88	0.645	16.62	0.97	0.66	2.23	725.06	710.02	695.28	1.95	33.70
26	PBW 752	882.00	521.00	361.00	0.588	20.41	0.87	0.59	2.50	701.50	677.88	655.06	1.87	40.93
27	DBW 71	848.50	547.25	301.25	0.594	17.04	0.92	0.64	2.29	697.88	681.43	665.37	1.88	35.50
28	WH 1123	903.13	696.75	206.38	0.805	11.67	1.17	0.77	1.92	799.94	793.25	786.63	2.19	22.85
29	WH 1266	753.00	606.38	146.63	0.584	8.29	1.01	0.81	1.84	679.69	675.72	671.78	1.87	19.47
30	WH 1239	977.63	560.38	417.25	0.701	23.60	0.94	0.57	2.58	769.00	740.16	712.40	2.04	42.68
31	WH 1184	933.75	620.13	313.63	0.741	17.74	1.04	0.66	2.23	776.94	760.95	745.29	2.09	33.59
32	WH 1097	719.88	468.50	251.38	0.431	14.22	0.78	0.65	2.27	594.19	580.74	567.60	1.60	34.92
33	WH 1260	914.38	612.63	301.75	0.717	17.06	1.03	0.67	2.21	763.50	748.44	733.69	2.06	33.00
34	WH 1156	986.00	562.00	424.00	0.709	23.98	0.94	0.57	2.60	774.00	744.40	715.93	2.06	43.00
35	WH 1263	1014.00	634.50	379.50	0.823	21.46	1.06	0.63	2.36	824.25	802.11	780.57	2.21	37.43
36	WH 1131	900.88	582.63	318.25	0.671	18.00	0.98	0.65	2.29	741.75	724.48	707.61	1.99	35.33
37	WH 1265	844.50	618.75	225.75	0.668	12.77	1.04	0.73	2.02	731.63	722.87	714.21	1.99	26.73
38	HD 3219	785.50	567.88	217.63	0.571	12.31	0.95	0.72	2.05	676.69	667.88	659.19	1.84	27.71
39	WH 1155	804.63	563.50	241.13	0.580	13.64	0.94	0.70	2.11	684.06	673.35	662.81	1.85	29.97
40	C 306	783.50	529.50	254.00	0.531	14.36	0.89	0.68	2.19	656.50	644.10	631.93	1.77	32.42
41	WH 1202	889.88	692.75	197.13	0.789	11.15	1.16	0.78	1.90	791.31	785.15	779.04	2.17	22.15
42	NBPGR 9	745.75	626.00	119.75	0.597	6.77	1.05	0.84	1.76	685.88	683.26	680.65	1.89	16.06
43	NBPGR 10	941.38	573.88	367.50	0.691	20.78	0.96	0.61	2.43	757.63	735.00	713.06	2.03	39.04
44	DBW 88	846.25	585.50	260.75	0.634	14.75	0.98	0.69	2.14	715.88	703.90	692.13	1.94	30.81
45	WH 1247	798.38	604.88	193.50	0.618	10.94	1.01	0.76	1.95	701.63	694.92	688.28	1.92	24.24
46	WH 1240	794.63	551.63	243.00	0.561	13.74	0.92	0.69	2.13	673.13	662.07	651.19	1.82	30.58
47	DBW 90	915.00	597.13	317.88	0.699	17.98	1.00	0.65	2.27	756.06	739.17	722.65	2.03	34.74
48	WH 711	838.00	524.13	313.88	0.562	17.75	0.88	0.63	2.37	681.06	662.73	644.90	1.82	37.46
49	NBPGR 35	889.75	556.63	333.13	0.634	18.84	0.93	0.63	2.37	723.19	703.75	684.83	1.94	37.44
50	WH 1021	914.50	683.75	230.75	0.800	13.05	1.14	0.75	1.98	799.13	790.75	782.47	2.18	25.23
Mean		884.17	597.53	286.65	0.68	16.21	1.00	0.68	2.20	740.85	726.01	711.53	2.00	32.11
Min		719.88	468.50	119.75	0.43	6.77	0.78	0.54	1.76	594.19	580.74	567.60	1.60	16.06
Max		1108.38	700.63	489.25	0.96	27.67	1.17	0.84	2.76	893.63	867.44	842.02	2.39	46.43

*STI* stress tolerance index, *MRP* mean relative performance, *HM* harmonic mean, *GMP* geometric mean, *MP* mean productivity, *Ys* grain yield of genotypes under heat stress condition, *YI* yield index, *Yp* grain yield of genotypes under normal condition, *YSI* yield stability index, *PYR* percent yield reduction, *RSI* relative stress index, *TOL* tolerance index, *SSPI* stress susceptibility percentage index.

### Correlation between grain yield and stress tolerance indices

The correlation coefficient between grain yield under normal and late sown conditions and heat tolerance indicators were calculated over the years to estimate the most appropriate stress tolerant criterion (Table 5).

**Table 5:** Correlation coefficient between grain yield (Yp and Ys) of wheat genotypes and stress tolerance indices.

	TOL	STI	SSPI	YI	YSI	RSI	MP	GMP	HM	MRP	PYR	Ys	Yp
TOL	1												
STI	0.354**	1											
SSPI	1**	0.354**	1										
YI	− 0.227*	0.828**	− 0.227*	1									
YSI	− 0.953**	− 0.104^NS^	− 0.953**	0.464**	1								
RSI	0.959**	0.088^NS^	0.959**	− 0.478**	− 0.982**	1							
MP	0.454**	0.992**	0.454**	0.765**	− 0.206*	0.197*	1						
GMP	0.354**	0.998**	0.354**	0.830**	− 0.104^NS^	0.088^NS^	0.994**	1					
HM	0.249*	0.992**	0.249*	0.885**	0.4^NS^	− 0.023^NS^	0.974**	0.994**	1				
MRP	0.353**	0.998**	0.353**	0.831**	− 0.099^NS^	0.088^NS^	0.994**	0.999**	0.992**	1			
PYR	0.953**	0.104^NS^	0.953**	− 0.464**	− 1**	0.982**	0.206*	0.104^NS^	− 0.4^NS^	0.099^NS^	1		
Ys	− 0.227*	0.828**	− 0.227*	1**	0.464**	− 0.478**	0.765**	0.830**	0.885**	0.831**	− 0.464**	1	
Yp	0.781**	0.859**	0.781**	0.431**	− 0.586**	0.582**	0.911**	0.860**	0.798**	0.860**	0.586**	0.431**	1

*, ** Significant at 0.05 and 0.01 levels of probability, respectively; *ns* not significant. For trait code description, refer to Table 2.

A positive significant correlation was found between Yp and Ys (0.431) indicating that they may be used to identify high-yielding genotypes under both conditions. Grain yield was negatively correlated with TOL, SSPI, RSI and PYR under stress (− 0.227, − 0.227, − 0.478 and − 0.464, respectively) but positively correlated under normal conditions (0.781, 0.781, and 0.582, respectively). Therefore, selection based on these indices will enhance grain production under non stressed conditions but decrease under stress conditions. YSI had a positive significant correlation with Ys (0.464), but a negative correlation with Yp (− 0.586). This is a more effective index to distinguish heat tolerant and susceptible genotypes. According to Nouri et al.^28^ YSI can be a useful index in distinguishing the higher and lower stability of genotyoes under stress conditions. The STI, YI, MP, GMP, HM and MRP had a high positive significant correlation with the grain yield (Yp and Ys). These indices were selected as the better ones, used to identify genotypes with high yield under both conditions. Based on these indices the genotype HD 2967 followed by WH 1249, WH 1142 and WH 1263 had identified as high yielding under both conditions. MP showed positive significant association with all indices except YSI. TOL and SSPI were also positively correlated with all indices except YI and YSI. The positive significant correlation of YI and YSI with Ys while negative with TOL and SSPI indicated that these two indices can be used to distinguish the genotypes that are stable and heat tolerant. The lowest value of TOL and SSPI can be used to select the genotypes that showed high yield under heat stress conditions and can be treated as heat tolerant genotypes. In this study the genotype WH 1266 followed by HI 1617 and WH 1025 had lowest TOL and SSPI.

### Principal component and biplot analysis

Principal component analysis (PCA) was performed using grain yield (Yp and Ys) and the heat stress tolerance indicators to identify stress tolerant genotypes (Table 6) Out of thirteen principal components (PCs), the first two components with an eigen value > 1.0 contribute maximum variation, i.e. 97.7%. The first (PC1) and second (PC2) principal components contributed to the total variations by 54.7% and 43% (Table 6). The first principal component (PC1) exhibited a high positive correlation with STI, MRP, HM and GMP (0.99). Among all the stress indices, STI, MRP, HM and GMP have highest variation. The second (PC2) principal component exhibited a high positive correlation with PYR, SSPI, PYR and RSI.

**Table 6:** Result of principal component analysis based on grain yield of genotypes and stress tolerance indices.

Components	PC1	PC2
Eigen value	7.11	5.59
Variance %	54.7	43
Cumulative	54.7	97.7
STI	0.99	0.06
MRP	0.99	0.08
HM	0.99	0
GMP	0.99	0.09
MP	0.96	0.22
Ys	0.89	− 0.43
YI	0.89	− 0.43
Yp	0.76	0.62
YSI	0.12	− 0.98
PYR	− 0.12	0.98
RSI	− 0.09	0.98
TOL	0.19	0.97
SSPI	0.19	0.97

*PC1* first principal component, *PC2* second principal component. For trait code description, refer to Table 3.

**Figure 2:**
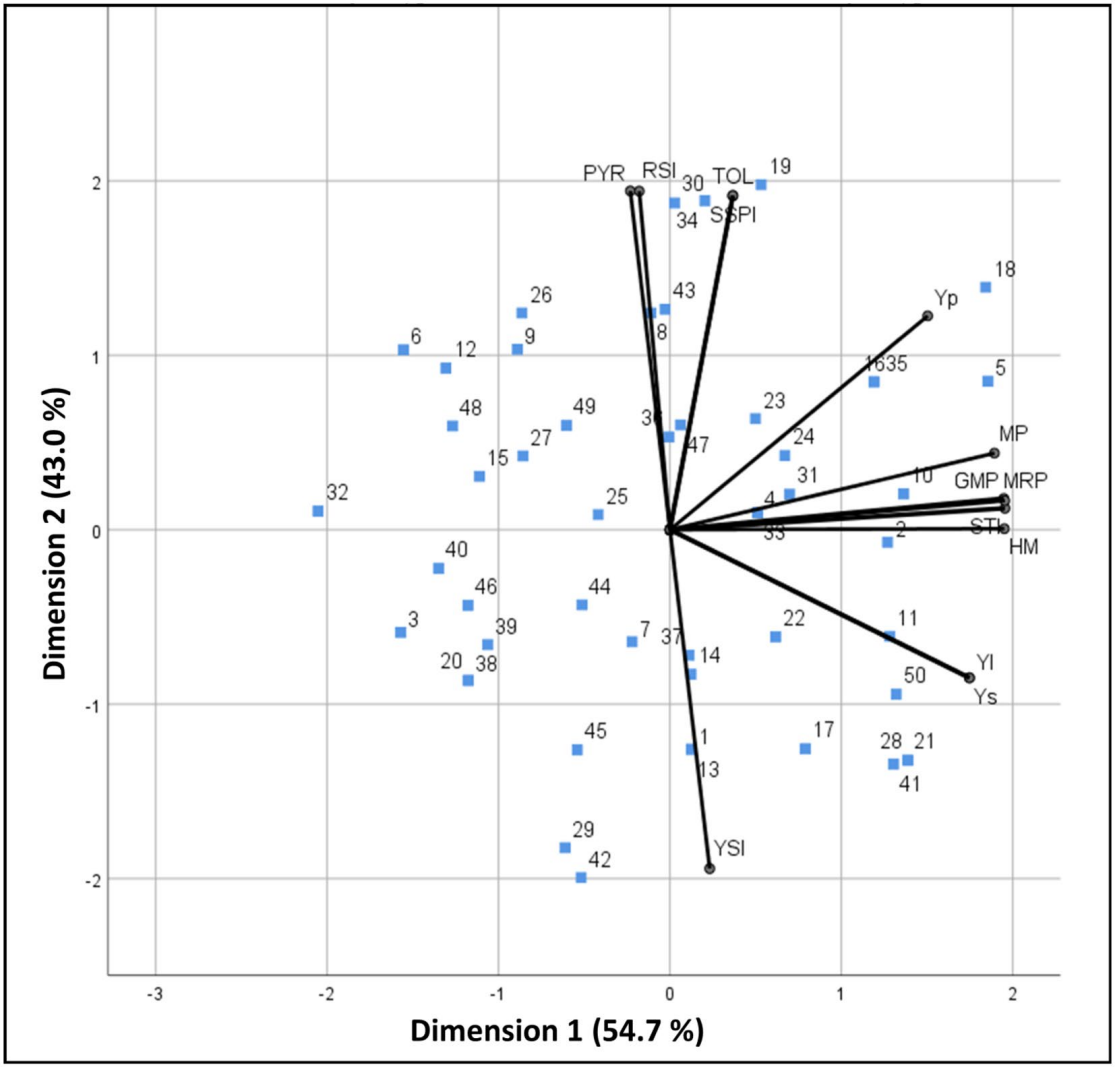
Biplot drawn based on PCA result showing correlation among traits. PC1 (Dimension 1) = First principal component, PC2 (Dimension 2) = Second principal component, STI = Stress tolerance index, MRP = Mean relative performance, HM = Harmonic mean, GMP = Geometric mean, MP = Mean productivity, Ys = Grain yield of genotypes under heat stress condition, YI = Yield index, Yp = Grain yield of genotypes under normal condition, YSI = Yield stability index, PYR = Percent yield reduction, RSI = Relative stress index, TOL = Tolerance index, SSPI = Stress susceptibility percentage index.

A biplot was constructed using PC1 and PC2 to compare genotypes and the correlations among heat tolerance indices (Fig. 2). Based on the greater PC1 but low PC2, wheat genotypes 11, 21, 28, 41 and 50 (HD 3086, HI 1617, WH 1123, WH 1202 and WH 1021) are stable under normal and stress conditions (Fig. 2). While genotypes 6, 9, 12 and 26 (WH 542, WH 1105, WH 283 and PBW 752) are less performing or heat susceptible under stress conditions as their PC2 is higher (positive) and PC1 is low (negative). The genotypes HD 2967 and WH 1249 had highest PC1 and influenced by stress indices viz., STI, MRP, MP and GMP. These genotypes were identified as most heat tolerant genotypes.

### Cluster analysis

**Figure 3:**
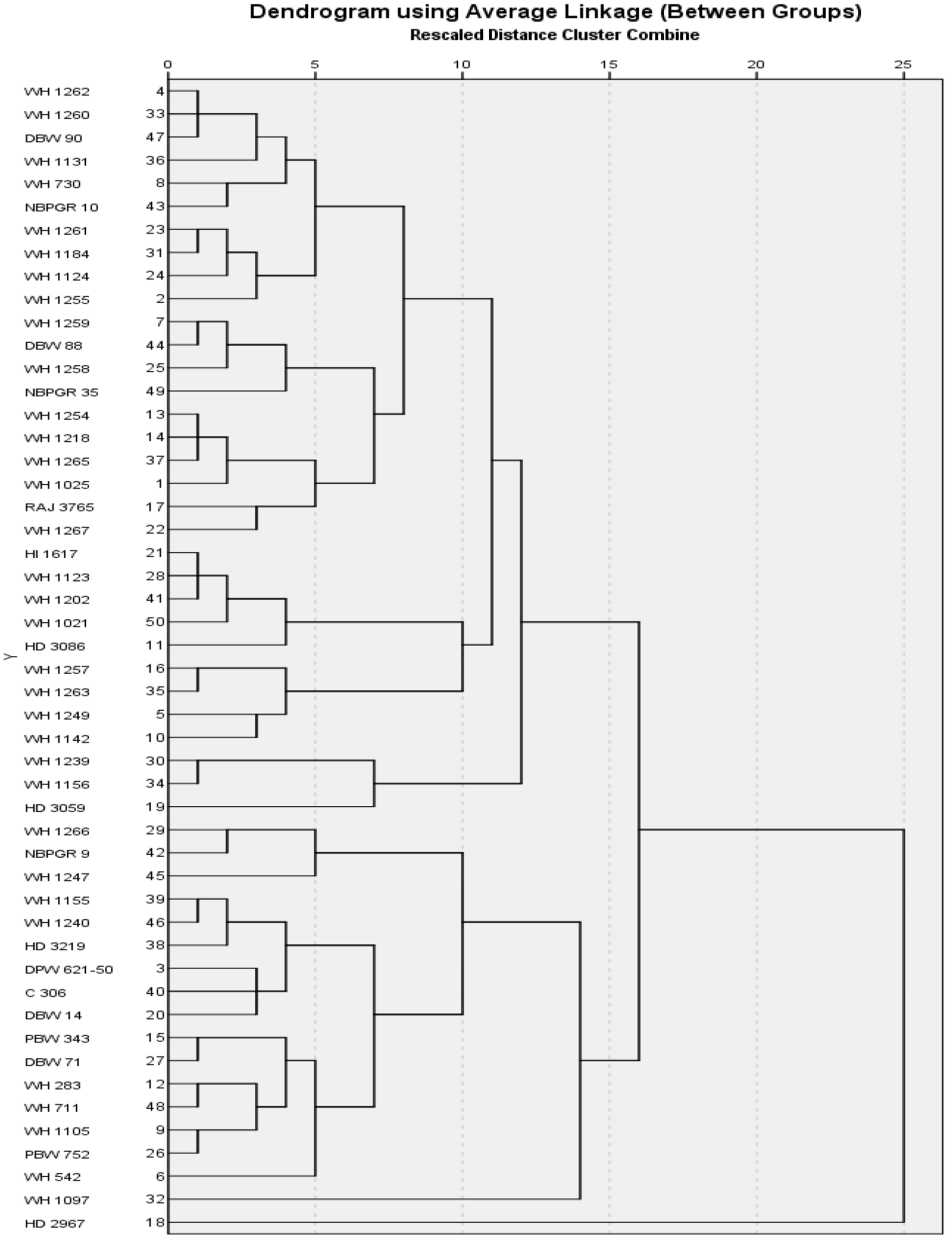
Dendrogram representing the clustering pattern of wheat genotypes based on heat stress indices.

Based on the heat tolerant indices, like MP, GMP, HM, STI and MRP, all studied wheat genotypes were clustered into seven groups (Table 7 and Fig. 3).

**Table 7:** Clustering of wheat genotypes using average linkage (between groups) method based on heat stress indices.

Clusters	No of genotypes	Cluster members
Cluster 1	20	WH 1262, WH 1260, DBW 90, WH 1131, WH 730, NBPGR 10, WH 1261, WH 1184, WH 1124, WH 1255, WH 1259, DBW 88, WH 1258, NBPGR 35, WH 1254, WH 1218, WH 1265, WH 1025, RAJ 3765, WH 1267
Cluster 2	9	HI 1617, WH 1123, WH 1202, WH 1021, HD 3086, WH 1257, WH 1263, WH 1249, WH 1142
Cluster 3	3	WH 1239, WH 1156, HD 3059
Cluster 4	3	WH 1266, NBPGR 9, WH 1247
Cluster 5	13	WH 1155, WH 1240, HD 3219, DPW 621–50, C 306, DBW 14, PBW 343, DBW 71, WH 283, WH 711, WH 1105, PBW 752, WH 542
Cluster 6	1	WH 1097
Cluster 7	1	HD 2967

The cluster I contained maximum genotypes (20) while cluster VI and VII had minimum one genotype in each. The genotypes belonged to same cluster had more similarities while genotypes among different clusters showed higher variations in values of stress tolerance indices. In this the highest value of MP, GMP, HM, STI and MRP possessed by genotype of cluster VII followed by genotypes belonged to cluster II, IV and I while minimum was exhibited by the genotype of cluster VI followed by genotypes of cluster III and V. Thus the genotypes (HD 2967) of cluster VII with highest value of tolerant indices was treated as highly heat tolerant genotype while the genotype (WH 1097) of cluster VI was as heat susceptible genotype.

## Discussion

In this study, stress indices were screened using grain yield and mean grain yield for analysis of heat tolerance in different wheat genotypes and also identified highly performing heat tolerant genotypes. High temperature significantly reduced the grain yield of wheat genotypes. Similar results were reported in other investigations^29,30^. Different wheat growing areas have varied stress level according to environment of that area^31^. Grain yield was negatively correlated with heat stress and it causes major problems for plant breeders to maintain high yield. Under late sown conditions wheat genotypes influenced at anthesis and post anthesis stage by a heat stress of approaximate more than 3–4 °C than normal sown genotypes.

Pooled ANOVA showed significant effects of heat stress on growth, development and finally on grain yield in wheat genoypes. Stress patterns can vary significantly between environments of different wheat-growing areas, showing a genotype by environment interaction. Furthermore, a substantial genotype by environment interaction was identified, which revealed that each genotype responded differently in two environments with regard to grain production and other yield parameters. The significance of mean sum of squares for all the stress indices for grain production in all genotypes, indicating the diversity of genotypes with high temperature tolerance^32^. Genotypes in different environments indicating presence of variation in the wheat germplasm for heat tolerance. On the basis of variability in cultivars, plant breeders used many approaches to identify and selection of high yielding genotypes under stress conditions. According to Basavaraj et al.^20^ one such approach is ANOVA to identify performance of genotypes using selection indices.

Several stress tolerance indices are efficiently exploited for the identification of tolerant genotypes under stress conditions. Stress tolerance index in wheat was used in various research to select genotypes that could withstand high temperatures^10,33,34^. Smaller values of TOL are preferred to select tolerant genotypes since larger values indicate a higher susceptibility to stress. The value of tolerance increases as SSPI and TOL values decrease, but they are unable to differentiate between genotypes that have higher yield in both conditions. When genotypes are selected based on TOL and SSPI, those with less yield performance under normal conditions and higher yield under stressful conditions are favoured. The highest values for TOL, RSI and SSPI belonged to HD 3059. This genotype produced high grain yields under normal condition and low yields in stress, it was classified as a heat sensitive genotype. The genotype NBPGR 9 exhibited the lowest TOL and PYR values, indicating that the yield difference between two conditions is minimal. Low TOL as well as grain yield should be taken in consideration for selection of high yielding genotypes. Dorostkar et al.^35^ and Kumar et al.^2^ revealed similar results in wheat genotypes under both conditions. According to Erdemci^36^ and Shabani et al.^37^ STI is a more appropriate parameter to screen tolerant genotypes of chickpeas. A genotype of superior performance in both normal and stressed environments is shown by higher values for STI, MP, and GMP. Furthermore, the genotypes chosen using STI will have higher grain yield and stress tolerance. The selections based on MP typically boost the average performance of genotypes in both stress and non-stress conditions and fail to make a distinction between stress-tolerant and high-yielding genotypes. MP favours higher yield potential and lower stress tolerance^2^. In our studies based on HM, MRP, GMP and STI genotypes HD 2967 had the highest values. The genotype HD 2967 was more productive under stress conditions than the remaining genotypes. Basavaraj et al.^20^ and Kamrani et al.^11^ also presented similar results and suggested higher yielding and heat tolerant genotypes could be selected on the basis of high values of MRP, GMP and STI. The genotype WH 1097 showed the lowest values for HM, MRP, GMP, MP and YI. The highest value for YSI was noticed in NBPGR 9. The genotype NBPGR 9 could be selected as a heat tolerant genotype as it represented lowest value for SSPI and highest value for YSI. Similar results were observed by Basavaraj et al.^20^ in rice and suggested that SSI and YSI could be used to identify higher yielding genotypes under stress conditions rather than under normal conditions. The highest values for YI belonged to genotypes HI 1617 and WH 1123, so on the basis of higher values for YI these genotypes are stress tolerant genotypes. According to Ashraf et al.^38^ and Singh et al.^39^ the line would be tolerant to stress conditions that had a higher value of YI.

A single approach based on values of different stress indices is not enough in selection of different heat tolerant or susceptible genotypes. Thus, to find the most suitable stress indices for heat stress tolerance, correlation co-efficient was analysed between grain yields (Yp and Ys) of both conditions and heat stress indices. The results of our study, Yp and Ys are positively correlated with each other matched with results of investigations reported by were reported by Kamrani et al.^11^ and suggested that based on this correlation, high performing genotypes can be identified under both conditions. Thus, the outcomes of the normal condition will be effective for selecting a heat-prone condition indirectly. Grain yield was negatively correlated with TOL, SSPI, and RSI under stress but positively correlated under normal conditions whereas YSI had a positive significant correlation with Ys, but a negative correlation with Yp. Poudel et al.^10^ found similar results and stated that TOL and SSI with lower values and YSI with higher values help us in selection of stress tolerant genotypes. The STI, YI, MP, GMP and HM had a high positive significant correlations with the grain yield (Yp and Ys). Ivi´c et al.^40^ found same correlations in nitrogen deficiency tolerant wheat genotypes and Jha et al.^41^ suggested that these indices can be used to identify the high yielding genotypes under both conditions.

PCA was done to determine the percent contribution of major components and indices to total variance, using grain yield under both conditions and heat stress tolerance indices. Although the correlation coefficient is appropriate for analyzing the relationships between two variables, many authors Nouri et al.^28^ and Talebi et al.^42^ had indicated that PCA is a better criterion than correlation coefficient for selection of the best yielding genotypes in normal as well as stress conditions. PCA shows the association between all traits at once and also decreases the number of traits that contribute to the maximum percentage of total variations. From the above result, it was concluded that the components whose eigen value was greater than 1 have higher variation than average. Therefore, it is considered as the basis for the selection of the components. The major variable in this study was yield, which was used to conduct the analysis. PC1 is positively associated with Ys, YI, MRP, HM, MP, GMP, STI, YSI and Yp. Thus, this component (PC1) can be called a “yield potential and heat tolerance component” under both the conditions. Similarly, PC2 is a strong association with SSPI, TOL, RSI and PYR and can be called a “stress susceptibility component”. The second component can be used to identify the heat susceptible genotype. Using same approach, correlation analysis Puri et al.^33^ and Kamrani et al.^11^ named first two principal components. Under normal and stress conditions highly performing genotypes have higher PC1 and lower PC2. According to Kaya et al.^43^ genotypes with higher PC1 but low PC2 are stable and vice versa.

In biplot analysis, cosine of the angle between their vectors indicate correlations among the indices^44^. Thus, two indices are positively correlated when the angle between two vectors is obtuse angle and negative correlation is shown by acute angle. When two vectors are perpendicular to each other, no correlation occurs between them. The biplot displayed that Yp and Ys exhibited positive associations with YI, HM, STI, MRP, GMP and MP while Ys was negatively correlated with TOL, SSPI, RSI and PYR as indicated by the obtuse and acute angles between their vectors, respectively (Fig. 2). GMP showed zero correlation with RSI as both are at 90°.

Based on the heat tolerant indices, like MP, GMP, HM, STI and MRP, all studied wheat genotypes were grouped into seven clusters. In this the highest value of MP, GMP, HM, STI and MRP was possessed by genotype of cluster VII followed by genotypes belonged to cluster II while minimum was exhibited by the genotype of cluster VI followed by genotypes of cluster Naghavi et al.^45^ also clustered the eight genotypes of maize into three classes by using stress tolerant indices like MP, GMP, STI and found that the genotypes with high value of these indices were stress tolerant genotypes which showed mean values were treated as semi tolerant to stress.Using grain yield (under both conditions) and stress tolerance indices. Thana et al.^46^ classified all studied genotypes in five clusters according to their performance and stress tolerance degree and found that a genotype with high values of MP, GMP, HM, STI and YSI is best performing and stress tolerant. So, genotypes with high values of MP, GMP, HM, STI and YSI might be used as parents in breeding programs to develop stress tolerant genotypes. Jha et al.^41,47,48^ generated different clusters of chickpea genotypes to select superior stress tolerance genotypes, based on various stress tolerance indices and other morphological traits and suggested that the genotypes belonging to distant group might be used in breeding programme for producing stress tolerance genotype in chickpea. They also found that the stress indices, viz. MP, GMP, YI and SSI could be used in breeding programme for selecting superior genotypes to sustain chickpea yield under stressed conditions.

## Conclusions

The current study used heat stress indices for assessment of 50 wheat genotypes grown in normal and stress environments concluded that heat stress adversely affects the performance in all genotypes under late sown as it decreases the yield. The results of correlation coefficient, PCA and biplot analysis exhibited the highly positive association of STI, MP, GMP, HM and MRP with Yp and Ys while TOL and SSPI negatively correlated with Ys. For cluster analysis same heat tolerance indices (STI, MP, GMP, HM and MRP) were used to differentiate the genotypes as heat tolerant or heat susceptible ones. Therefore, these stress indices were found to be the better predictors for the selection of desirable high yielding genotypes under both conditions. Thus stress indices could be better approaches for the selection of high temperature tolerant genotypes from both environments. Based on the contribution of these different indices, genotypes, namely, HD 2967, WH 1249, HI 1617, WH 1202, WH 1021 and WH 1142 were identified as tolerant and high performing genotypes in both environments. As a result, these heat-tolerant genotypes can be employed as genetic resources in agricultural enhancement programmes.

## Supplementary Information

Supplementary Table S1.

## Data Availability

All the data are presented in the body of the manuscript or the supplementary material.

## Abbreviations

ANOVA: Analysis of variance
GMP: Geometric mean
HM: Harmonic mean
MP: Mean productivity
MRP: Mean relative performance
NS: Not significant
PC1: First principal component
PC2: Second principal component
PYR: Percent yield reduction
RSI: Relative stress index
SSPI: Stress susceptibility percentage index
STI: Stress tolerance index
TOL: Tolerance index
YI: Yield index
Yp: Grain yield of genotypes under normal condition
Ys: Grain yield of genotypes under heat stress condition
YSI: Yield stability index
