# Supplementary material for: Heat stress tolerance indices for identification of the heat tolerant wheat genotypes
Source: Sci Rep. 2023 Jul 5;13:10842. doi: 10.1038/s41598-023-37634-8 (PMC10322891; doi:10.1038/s41598-023-37634-8)
Supplement: Supplementary file 1 — Supplementary Table S1. [file 41598_2023_37634_MOESM1_ESM.docx]

**Table S1:** **Pooled weekly weather parameters [minimum and maximum temperature (^0^C), relative humidity (morning and evening), bright sun shine hours and rainfall (mm)] during wheat growing season (2018-19 & 2019-20).**

| Pooled weather parameters 2018-19 & 2019-20 | | | | | | |
| --- | --- | --- | --- | --- | --- | --- |
|  | Temperature (°C ) | | Relative humidity | | Bright sun shine hours | Rainfall (mm) |
|  | Max | Min | M | E |  |  |
| Nov3-9 | 27.90 | 11.40 | 87.50 | 38.65 | 4.95 | 0.15 |
| Nov10-16 | 27.15 | 12.70 | 88.70 | 47.00 | 3.00 | 0.00 |
| Nov17-23 | 27.05 | 10.90 | 87.80 | 43.05 | 5.25 | 0.00 |
| Nov 24-30 | 24.90 | 10.75 | 92.60 | 54.35 | 4.15 | 6.00 |
| Dec1-7 | 24.00 | 6.75 | 92.15 | 46.10 | 5.65 | 0.00 |
| Dec8-14 | 20.10 | 8.00 | 92.70 | 65.40 | 2.90 | 2.25 |
| Dec15-21 | 17.20 | 4.05 | 96.20 | 65.35 | 3.70 | 0.00 |
| Dec22-28 | 15.85 | 2.25 | 95.30 | 61.95 | 3.05 | 0.00 |
| Dec29-Jan4 | 18.10 | 5.70 | 95.60 | 62.95 | 3.40 | 3.65 |
| Jan5-11 | 18.50 | 5.65 | 94.70 | 61.85 | 3.85 | 1.60 |
| Jan12-18 | 16.90 | 4.80 | 95.10 | 68.50 | 3.55 | 0.00 |
| Jan19-25 | 18.70 | 4.90 | 97.80 | 59.55 | 5.25 | 6.85 |
| Jan26-Feb1 | 17.95 | 4.60 | 96.85 | 63.00 | 5.10 | 0.00 |
| Feb2-8 | 20.55 | 4.85 | 92.05 | 50.75 | 6.45 | 0.00 |
| Feb9-15 | 22.35 | 7.25 | 93.30 | 51.50 | 5.90 | 0.00 |
| Feb16-22 | 23.00 | 9.75 | 89.40 | 55.35 | 6.00 | 5.45 |
| Feb23-29 | 23.45 | 10.05 | 93.75 | 54.10 | 6.25 | 7.50 |
| Mar1-7 | 23.65 | 10.10 | 89.20 | 52.10 | 6.95 | 30.90 |
| Mar8-14 | 24.35 | 9.45 | 92.30 | 50.75 | 6.30 | 5.80 |
| Mar15-21 | 29.05 | 13.10 | 85.20 | 47.05 | 6.75 | 0.75 |
| Mar22-28 | 30.10 | 14.25 | 86.35 | 41.60 | 6.25 | 10.30 |
| Mar29-Apr4 | 33.50 | 14.70 | 77.30 | 32.75 | 7.95 | 3.65 |
| Apr5-11 | 36.00 | 18.85 | 71.00 | 25.50 | 7.20 | 0.00 |
| Apr12-18 | 34.50 | 18.00 | 75.85 | 31.10 | 7.65 | 4.50 |
